# Do the Fittest Really Survive?

**Published:** 1879-07

**Authors:** Ausburn Towner


### DO THE FITTEST REALLY SURVIVE ?

AUSBURN TOWNER.

I find no more delightful manner of spending some of the few leisure minutes that fall to my lot, than looking through the old bookstands in New York that you meet with in many unexpected places in the neighborhood of Chatham Street and adjacent thoroughfares. I have chanced upon many a volume that seemed to me to be a treasure, full of matter that deserved rather to be perpetuated than covered deep with the dust of forgetfulness. I have, on my shelves, rows of books picked up in this way at a cost hardly appreciable and yet representing so much real value in my eyes, that I would be loth to part with them if the cents they cost, were made into as many dollars. Comparing them with many of the works that have withstood the advance of time and have been embalmed and preserved as standard, the thought that is expressed in the title of this article: "Do the fittest really survive ?" has often crossed my mind.

There is no method by which, in the limits allowed in the pages of the BISTOURY, an intelligent comparison can be made between some of the forgotten books and those that, in some unaccountable manner, have been preserved from oblivion. If I should say: Compare the novel "John DeCastro" with any one of Sterne's or Richardson's or Fielding's; "King

Rene's Daughter" with "Marmion" or any of Tupper's works, or "The Dead Marquise" with "Helen's Babies" or "Uncle Tom's Cabin," it would only be meaningless to the average reader, for although he is familiar, at least, with the names of Richardson and Scott and Tupper, "John DeCastro," "King Rene," and "The Dead Marquise," he now doubtless hears of, for the first time. But I am sure, if the comparison should be made, not only would the inquiry that I have put at the head of this, involuntarily spring to the mind, but the further question would arise: Upon what rules are founded the immortality of literary work, that oftentimes, the poorest lives?[1]

I know of no more thorough method of showing at least how curiously public sentiment acts in this matter, than to take one of the so-called English classics and exhibit it at its true and genuine worth. The same remark might be made of most of the books written prior to this century, that they were very well for the times in which they appeared, and beyond that, it might do to preserve a sample or two merely as objects of curiosity, but by no means as models or as examples of what the coming generations may imitate but can never equal. I never hear the platitudes and cant concerning old writers, Homer, Virgil, Horace, Milton, Shakespeare, Rosseau, Voltaire, Addison or any other of these worthies, without thinking how absurd the assertion that they can never be equaled, to say nothing of surpassing them! You might as well contend that the tallow dip and the whale oil lamp of a generation or so back, were better than the modern contrivances for lighting our streets and houses; that the world has never made any progress at all; that the mental faculties have not been improved; that while all of men's surroundings have advanced and a nearer approach to excellence in every other way has been made, the literary faculties have remained stationary, nay, worse than that, have retrograded, for the mind cannot stand still; it must either go forward or backward. I, for one, am not agreed to such assumptions. Better work in all lines of mental endeavor is done every day than any that preceded it. Due credit should be accorded to the men whose names I have set down and to many others, but they by no means outshine so completely as some would have us believe, the others who have followed them. Howe invented the needle that made the sewing machine possible, but he is not therefore a greater man than the many who followed him and improved on his invention. Prof. Morse discovered a way to bottle up electricity, and make it do his bidding, but his fame is not so great, nor should it be, that it can eclipse that of House or Edison or the multitude of other electricians who have improved on what he discovered. The earlier poets and writers, who found out the pleasure that is given by fictitious narratives, the beauties of a rhythmical disposition of words or the ear tickling music of rhyme, certainly deserve to be remembered as long as the language is, but not more so than those who followed afterward and improved on their discoveries.[2]

As I have said, it would be well to take one of the conspicuous works of old and show what it really is, and I have selected Hudibras for this purpose, since it will do as well as any other. Its name is well known and quotations from it are frequent. It is published in numerous editions and styles and can be found in every well-appointed library in the land, among the standard works. It may be said that it is read very little. Very true, but the same remark can be made concerning Paradise Lost, Chaucer, Shakespeare, Burton or Boswell, and no library is considered complete unless these are in it.

What then are the claims that Hudibras has to maintain its place on the book shelves of the present day? It was first published 217 years ago next November, and is a satire in the interest of the court of Charles II of England, directed against the Puritans, Presbyterians and Roundheads generally[3] Now there is nothing

1—As for example, Bret Harte's "Heathen Chinee" poem, which will be remembered after his meritorious work has been forgotten, or Mrs. Sarah J. Hale's "Mary had a Little Lamb," that is the only enduring work of a literary life of more than three-quarters of a century.

2—It is a subject of amusement to me to observe the veneration, not to say adoration, bestowed upon Shakespeare, by many so-called philosophers, and mouthing actors who have graduated from the Bowery or in some Variety Hall. To one who reads with intelligence and thinks at all, and who is not blinded by what some one else has had to say on the subject, it is the veriest cant and rubbish. The plays that bear his name might do well enough for the times in which they are said to have been written, when actors, as a class, held about as high a position in society as tramps do in these days; when stage scenery was labeled: "This is a tree," or "This is a castle," or "This is a bridge," to be recognized, but they do not suffice in this generation. This is demonstrated more and more clearly every day. It is a sure loss to a manager to put on, no matter in what style, a Shakesperian play. The hard, common sense of the public is rapidly showing the true value of works that have no real foundation for their assumed excellence, except the dictum of some self-appointed critic.

3—I make this explanation in full, because although it is safe to assume that every one of my readers has heard of Hudibras, no one in one hundred of them know what it is all about.

truer concerning all literary efforts of this nature than that satire, as satire, with nothing else to sustain it, loses its whole force and point when the abuse at which it aims or the persons whom it attacks have ceased to exist or are forgotten. It then falls meaningless and dead upon the understanding, and no amount of explanation or number of foot-notes will make it of interest to the reader. Petroleum V. Nasby's letters are a case in point, and they are, comparatively, of so recent a date that the illustration is peculiarly an apt one. They had nothing but their bitter satire of President Johnson and his swinging 'round the circle, to lift them into notoriety. They were received, as probably most of my readers can recollect, with a hurrah by the whole public and were actually devoured with a ravenous appetite by a large majority of the reading people of our country. It would hardly be a profitable venture for a publisher to issue the letters in a book form now; the *nom de plume* is almost forgotten and a new generation reading them could but little understand their drift. They were timely and fitted the days in which they appeared. That was all. They could not live and did not deserve to live. Satires that have something behind them besides their venom, sometimes live. "Gulliver's Travels" is a satire and was a bitter one in its day, but the story it tells is a peculiar and entertainining one and has lived for itself outside of the satire. Pope's "Dunciad," Byron's "English Bards and Scotch Reviewers," and even Lowell's "Fable for Critics," are already losing their force as satires as we get farther and farther away from the men and times they concerned, but their splendid versification and genuine poetry still attract attention to them.[4]

Now the fight of King Charles' time is over and done, and those whom Butler satirised certainly did not lose. What he said cannot be appreciated in these days. Who cares about it? Every allusion needs a foot-note to explain it, and after the explanation the reader is not at all the wiser. It is safe to affirm then that it is not the satire of the book that gives it a place in the libraries of to-day.

As to the story, it concerns a certain impossible English Knight, Sir Hudibras,[5] and his equally impossible esquire Ralpho.[6] They go forth armed and equipped and engage in an encounter with a bear and a fiddler whom they capture and imprison. Then a rabble come and attack them and the two themselves are taken prisoners. A lady appears who pretends to be enamored of the Knight, obtains his release from jail and gets him into other trouble that he flees from. He sets about suing her, perhaps for breach of promise, but writes her a letter instead, which she answers and the book ends. The statement of the story is sufficient to show that, judged by it alone, the book is not worth the paper upon which it is printed.

Oftentimes the construction of a story is its chief merit, and is able to redeem it from contempt. On a more slender thread than this of Hudibras some of the most delightful compositions have been strung, but none so clumsily and worthlessly as this. It is called a Poem. If it is correctly classed I would like to know what to call "The Bride of Abydos" or "Lalla Rookh." Hudibras is not a Poem in any sense of the term, it is not even verse. It is simply dull prose printed hap hazard with a capital letter at the beginning of each line. Most of the lines have eight syllables, but no attention is paid to rhythmical combination or emphasis, and they therefore halt and hesitate worse than a lame horse. Nor can the work be accurately said to be made up of rhymes. There are consecutive couplets, one after another, page after page, that rhyme no more than does blank verse, although they are claimed to be rhymes. Including the arguments that precede each canto there are 11,510 lines in the story and thus 5,755 couplets. Of these 2,297 couplets do not rhyme at all. What would be said of any one setting up for a poet these days who should soberly put down for rhymes such words as speech and which, heathen and brethren, further and order, drumbeat and combat, and more than 2,000 others similar in sound? To even unattuned ears, the jingle of rhymed words is very pleasing but Hudibras has not even this to commend it.

It is claimed that the satire is so full of nature and life that it has furnished numerous quotations[7] that have become almost proverbs;

4—Rabelais' works are also satires, and I am afraid that all that has kept them alive is their extreme vulgarity.

5—I have heard a great many different pronunciations of this name, but it is excusable, for Butler himself was at loose ends in regard to it. He makes it rhyme with was, has, case, place, as and ass, so the reader can call it as he likes, only by no means Hudibrah!

6—Butler has the same difficulty in pronouncing this name, omitting the final *o* at pleasure and making it rhyme with half, save, have and safe indiscriminately.

7—Some of the best known of these are as follows:

"He could distinguish and divide
A hair 'twixt south and southwest side;
On either side he would dispute that it is full of sentiments that curtly expressed deserve to be preserved. Very well, granted, but is it worth while to dig through a hundred tons of dirt to find one grain of gold, especially when there are mines that will yield a much larger return? Let us save the gems if we so choose, but throw away the dross and not deify a toad, because it may possibly have a jewel in its head.

Possibly one can find some good in the book, the profusion of foot notes that its forgotten allusions make necessary. They are especially rich and show the editor to be a man of patient research, great learning and more than ordinary wit. After the arid desert of the text itself they come like a drink of cool water to a parched throat. If I was to advise, I should say read the foot notes and skip the alleged poem.

After this brief examination, that might be indefinitely amplified and elaborated, of this famous, co-called classic, I can conjure up but one excuse for its continued existence, and that, the same that makes museums of antiques interesting, to show with what really inferior things those who have gone before us, were satisfied. Or like the Sphinx or Pyramids, that whatever may have been their uses at one time, serve no purpose at all now except to stare at. Viewed in this light, let Hudibras and the multitude of other ancient books I could name, continue to live, but do not give them a position in advance of the productions of our own day and generation, for they by no means deserve it.[8]

Confute, change hands and still confute."
"For he could coin or counterfeit
New words with little or no wit."
"And we are fine cobwebs fit for o skull
That is empty when the moon is full."
"And prove their actions orthodox
By apostolic blows and knocks."
"Compound for sins they are inclined to
By damning those they have no mind to."
"For rhyme the rudder is of verses
With which, like ships, they steer their courses."

Butler's helm must have been sadly out of order.

"Like him who took the doctor's bill
And swallowed it instead o' th' pill."
"For brevity is very good
When we are or are not understood."
"For whatsoever we perpetrate
We do but row; we're steered by fate."
"Great actions are not always true sons
Of great and mighty resolutions.
"Your pettifoggers damn their souls
To share with knaves in cheating fools."
"Doubtless the pleasures is as great
Of being cheated as to cheat.
"Honor is like a widow won
With brisk attempt and putting on,
With ent'ring manfully and urging
Not slow approaches like a virgin."
"She that with poetry is won
Is but a desk to write upon."

8—There is one good story very poorly told that is worth copying, as it shows all of the metrical defects of Hudibras, and leaves on the mind of the reader an impression that the matter in the hands of a good poet could have been made excellent. It is as follows:

"Justice gives sentence many times
On one man for another's crimes.
Our brethren of New England use
Choice malfactors to excuse,
And hang the guiltless in their stead,
Of whom the churches have less need.
As lately 't happened in a town
There lived a cobler, and but one,
That out of doctrine could cut use,
And mend men's lives as well as shoes.
This precious brother having slain
In times of peace an Indian,
Not out of malice, but mere zeal,
Because he was an infidel,
The mighty Totti-poti-moy
Sent to our elders an envoy,
Complaining severely of the breach
Of league held forth by brother Patch
Against the articles in force
Between both churches, his and ours,
For which he craved the saints to render
Into his hands or hang the offender.
But they maturely having weighed
They had no more but him of the trade.
A man that served them in a double
Capacity to preach and cobble,
Resolved to spare him; yet to do
The Indian Hoghan Moghan too
Impartial justice, in his stead did
Hang an old weaver that was bed-rid.

